# Low Vitamin B12 Levels and Its Association With Insulin Resistance: A Potent Cardiovascular Risk Indicator in Childhood Asthma

**DOI:** 10.7759/cureus.39422

**Published:** 2023-05-24

**Authors:** Arulvijayavani Subramaniam, Sathiya Ramasamy, Soundararajan Palanisamy, Athisankaran Punniyamurthy, Sathishbabu Murugaiyan

**Affiliations:** 1 Biochemistry, Jawaharlal Institute of Postgraduate Medical Education and Research Karaikal, Karaikal, IND; 2 Biochemistry, Mahatma Gandhi Medical College and Research Institute, Puducherry, IND; 3 Paediatrics, Mahatma Gandhi Medical College and Research Institute, Puducherry, IND

**Keywords:** cardiometabolic risk, insulin resistance, pulmonary function test, vitamin b12, asthma

## Abstract

Introduction

As insulin resistance metabolically affects the body mass index (BMI), obese asthma children have more severe diseases than children with normal body mass index. A low level of vitamin B12 (Vit B12) is a known atherogenic factor by increasing the homocysteine level and therefore promotes cardiovascular morbidity and mortality. Limited studies have evaluated the role of serum B12 and insulin resistance among poorly controlled asthma in children. The purpose of the study was to compare the cardio-metabolic risk factor such as BMI, waist-hip ratio (WHR), insulin resistance, and vitamin B12 in well-controlled and poorly-controlled asthma patients and to determine the relationship between these parameters with the severity of asthma as assessed by Pulmonary Function Test.

Methodology

Based on the asthma control questionnaire and Global Initiative for Asthma (GINA) criteria, chronic asthma patients (n=60) of age 10-15 years were divided into two groups, namely well-controlled and poorly-controlled (30 each). Anthropometry was assessed by BMI and waist-hip ratio, and fasting blood samples were collected for the estimation of blood glucose, insulin, and serum vitamin B12 levels. Insulin resistance (HOMA-IR) was calculated using the formula- fasting glucose (mg/dL) x fasting insulin (µIU/mL)]/405. Forced expiratory volume (FEV1), forced vital capacity (FVC), and FEV1/FVC ratio were measured to assess the pulmonary function test.

Results

There were significant differences in the values of the BMI, insulin resistance, vitamin B12, and pulmonary function tests between poorly controlled and well-controlled asthma (p<0.01). The FEV1: FVC% was negatively correlated with BMI (r=0.53), WHR (r=0.50), glucose (r=0.68), insulin (r=0.68), Insulin resistance (r=0.80), and positive correlation with Vit B12 (0.73). In addition, Vit B12 and HOMA-IR correlate negatively (r=0.76).

Conclusion

This study concludes that the level of Vit B12 is decreased and insulin resistance is increased in poorly controlled asthmatic children in comparison to well-controlled asthma. These factors along with the increased BMI in poorly controlled asthma can predispose to cardiometabolic risk which needs attention.

## Introduction

Asthma and cardiovascular diseases are increasing in most countries, especially its incidence among children is considered a serious health problem. Uncontrolled chronic airway disorder causes limitations in daily activities and can be fatal too. Asthma is a chronic inflammatory condition associated with hypoxia, decreased in physical activity, and increased stress. Healthcare costs lost productivity due to absenteeism, and reduced participation in school life increase the burden still more. The prevalence of asthma in India is 24 per 1000 population accounting third of the world's asthmatics [1]. Adequate control of asthma symptoms is essential; if not controlled, may eventually lead to heart failure in the early part of life. Obesity is an inflammatory condition associated with insulin resistance, hypertension, diabetes mellitus, polycystic ovarian diseases, etc. Studies have also documented the role of insulin resistance in the causation of allergic asthma among obese children [2,3]. Children with obesity have an increased risk of developing asthma and obese children with asthma have a poor quality of life as assessed by frequent exacerbation of asthma, increased severity of symptoms, and difficulty in controlling their symptoms than children with normal weight [1,4,5]. 

Studies have reported that asthma and atherosclerosis are chronic comorbid diseases with lipo-metabolic cross-talk [5,6]. Vitamin B12 is involved in the metabolism of homocysteine and is linked to cardiovascular diseases. Studies have documented that COPD patients have poor B complex vitamin status, which has been implicated in unresponsive chronic cough [7,8]. A study has reported a statistically significant negative correlation between Vitamin B12 levels and insulin resistance among obese children [9]. Homeostasis model assessment of insulin resistance (HOMA-IR) has been evidenced to be a surrogate marker to assess insulin resistance and was documented to be a predictor for cardiovascular morbidity and mortality [10]. Hence, the present study was planned to compare the cardio-metabolic risk factor as assessed by BMI, waist-hip ratio (WHR), insulin resistance, and vitamin B12 in well-controlled and poorly-controlled asthma patients and to correlate these parameters with the severity of asthma as assessed by Pulmonary Function Test (FEV1, FVC) in poorly controlled asthma patients. This article was previously presented as a meeting abstract at the 48th National Conference of Association of Clinical Biochemistry of India on November 24, 2022.

## Materials and methods

This study was done by the Department of Biochemistry in collaboration with the Department of Pediatrics in a tertiary care center in South India for a period of three years (December 2018- December 2020). The study was approved by the research committee and Institute Ethics Committee - Human Studies (Faculty project/2018/06/25). A written informed consent was obtained from the parents and assent had been obtained from all the study subjects. Chronic asthma patients of the age group 10-15 years of age irrespective of the disease duration were recruited for the study. The participants filled out an asthma control test, including four symptom/reliever questions and the level of asthma control as self-assessed by the patients. They were divided into well-controlled (Asthma control test score of 20-25) and poorly-controlled asthma patients (Asthma control score of 5-15) according to the global initiative for asthma management (GINA) [11]. Thirty subjects were included in each group-well controlled and poorly controlled. Thirty age and sex-matched healthy children with normal pulmonary function tests were recruited as control subjects. Subjects with emphysema, bronchiectasis, infective bronchitis, allergic bronchitis, known diabetes of any type, hypertension, coronary heart diseases, cyanotic heart diseases, renal failure, gout, an endocrine disorder, patient on drugs other than anti-asthmatic agents, children with vitamin B12 supplementation within six months before enrolment in the study were excluded from the study.

Anthropometric measurements like height, weight, waist circumference, and hip circumference were measured. Body mass index was calculated using the formula weight in Kg/height in meter square. The waist-hip ratio was also calculated for all the study participants. Pulmonary function tests as assessed by forced expiratory volume (FEV1), forced vital capacity (FVC) and FEV1/FVC ratio were measured using a portable spirometer - MIR Winspiro spirobank II. Two milliliters of fasting venous blood sample was collected after taking aseptic precautions. The sample was centrifuged at 3500 rpm for 15 min and serum was separated. Fasting plasma glucose was estimated using glucose-oxidase and peroxidase methods in a fully automated chemistry analyzer immediately and the remaining sample was stored at -20° for further analysis. Vitamin B12 and Insulin levels were estimated using an electrochemical luminescence immunoassay in an E-Cobas analyzer (Cobas e11; Roche Diagnostics, USA). Insulin resistance was calculated using Homeostatic Model Assessment for Insulin Resistance (HOMA-IR) using the formula (fasting glucose(mg/dL) x fasting insulin(MIU/L))/405 [12].

Statistical analysis

Descriptive statistics were used to analyze the data. The normality of the data was checked using the Shapiro-Wilk test. The study parameters were non-normally distributed and were expressed as median with interquartile range. Differences between the study groups were analyzed using the independent value Kruskal-Wallis test. The correlation between the parameters and the pulmonary function test was analyzed using the Spearman correlation test. All statistical analysis was carried out at a 5% level of signiﬁcance for two-tailed signiﬁcance using SPSS software version 20 (IBM Corp., Armonk, NY).

## Results

The mean duration of asthma in well-controlled patients was six months (3 -15 months) and in poorly controlled patients were eight months (3.5- 19 months). Poorly controlled asthma subjects were obese and had more body mass index, and waist-hip ratio when compared with well-controlled asthma subjects. Pulmonary function test, assessed by spirometry, was low in poorly controlled asthma. Though the glucose levels were within the normal range in poorly controlled asthma subjects, it was increased when compared to well-controlled asthma subjects. Insulin levels were increased and insulin resistance was seen in children with poorly controlled asthma when compared to well-controlled asthma. Vitamin B12 levels were low in poorly controlled asthma subjects when compared to well-controlled asthma subjects (Table 1).

**Table 1:** Comparison of study parameters between well-controlled and poorly-controlled asthma patients * p < 0.05 statistically Significant FEV- Forced expiratory volume FVC -Forced Vital Capacity HOMA - IR - Homeostatic Model Assessment of Insulin Resistance

Parameters	Control (N = 30)	Well-controlled asthma (N = 30)	Poorly-controlled asthma (N = 30)	P value
Age in yrs	12 (11-14)	13.5(12-14)	13(12-14)	0.37
Body mass index	22.05 (21.42-23.43)	21.0 (20.5-21.25)	26.01 (25.05-26.68)	0.001*
Waist hip ratio	0.83 (0.80-0.87)	0.84(0.83-0.85)	0.91(0.89-0.92)	0.001*
FEV1 % predicted	92.4 (90.38-93.93)	81.65 (79.55-85.90)	60.75 (59.15-62.95)	0.001*
FVC % predicted	97.4 (96.7-99.32)	93.70 (91.3-96.7)	82.8 (82.33-85.95)	0.001*
FEV1: FVC %	94.92 (92.63 -97.53)	88.06 (85.0-90.37)	73.27 (68.65-76.79)	0.001*
Serum fasting Glucose (mg/dl)	72.5 (70.75-80.25)	80.00 (70.00-83.00)	107.0 (99.75-116.25)	0.001*
Serum Insulin (mIU/L)	10.0 (8.75-15)	18.0 (15.0-20.0	22.5 (19.0-24.25)	0.001*
HOMA - IR	2.13 (1.56-2.74)	3.46 (2.93-4.08)	5.74 (4.90-6.53)	0.001*
Vit B12 (pg/mL)	442.0 (418.0-551.75)	378.5 (252.5-432)	175.0 (162.25-190.0)	0.001*

**Figure 1:**
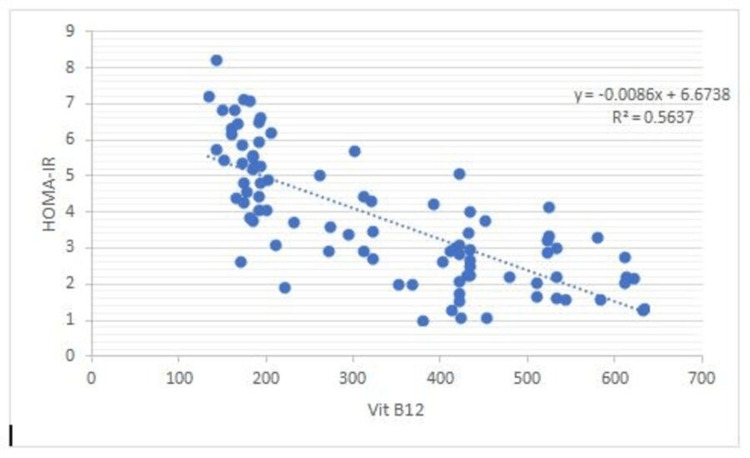
Association between vitamin B12 and insulin resistance Shows a negative correlation between vitamin B12 and insulin resistance (HOMA-IR)

**Figure 2:**
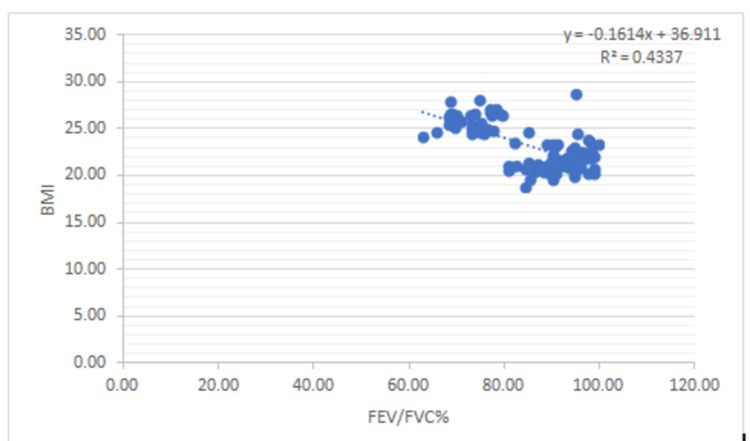
Correlation between FEV/FVC% and BMI Shows a negative correlation between FEV/FVC% and BMI FEV- Forced expiratory volume FVC- Forced vital vapacity BMI- Body mass index

**Figure 3:**
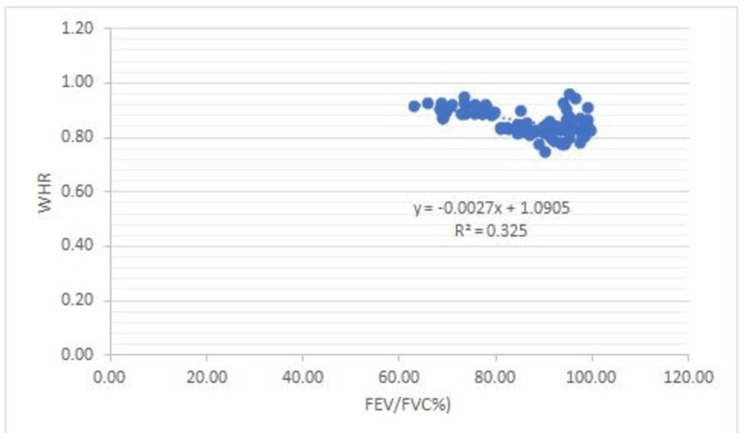
Correlation between FEV/FVC% and WHR Shows a negative correlation between FEV/FVC% and WHR FEV- Forced expiratory volume FVC- Forced vital Capacity WHR- Waist-hip ratio

**Figure 4:**
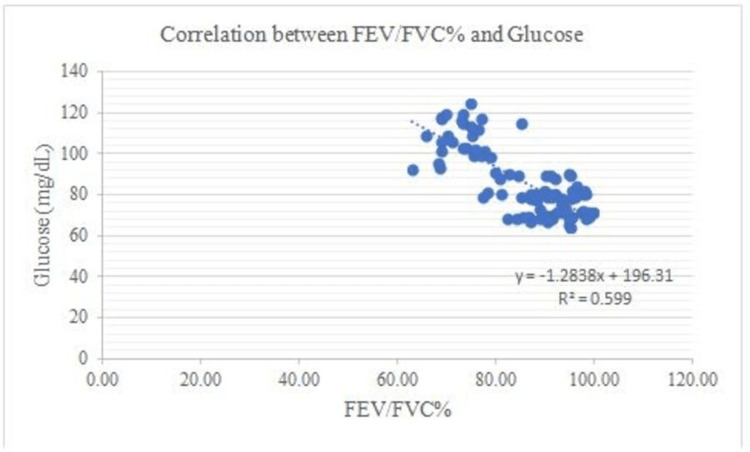
Correlation between FEV/FVC% and Glucose Shows a negative correlation between FEV/FVC% and Glucose FEV- Forced expiratory volume FVC- Forced vital capacity

**Figure 5:**
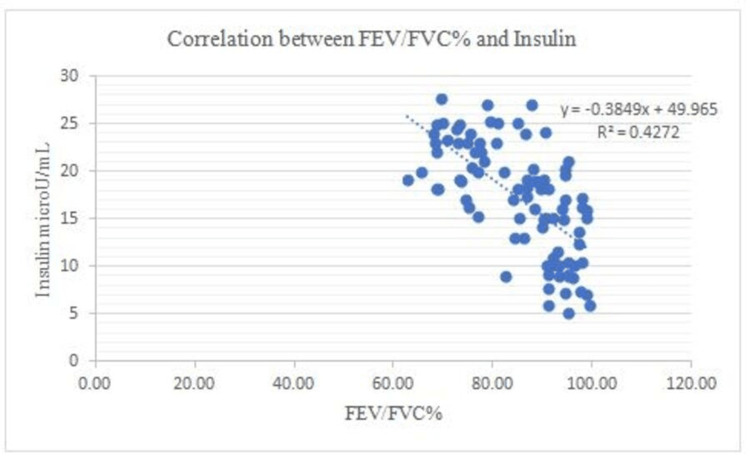
Correlation between FEV/FVC% and Insulin Shows a negative correlation between FEV/FVC% and Insulin FEV- Forced expiratory volume FVC- Forced vital capacity

**Figure 6:**
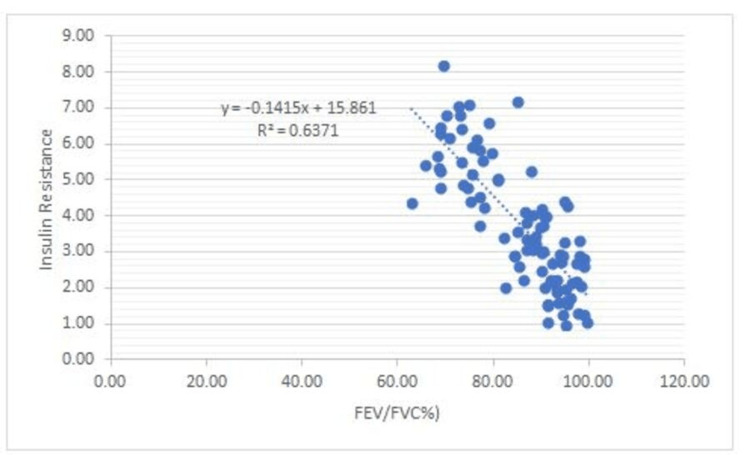
Correlation between FEV/FVC% and Insulin Resistance Shows a negative correlation between FEV/FVC% and Insulin Resistance FEV- Forced expiratory volume FVC- Forced vital capacity

**Figure 7:**
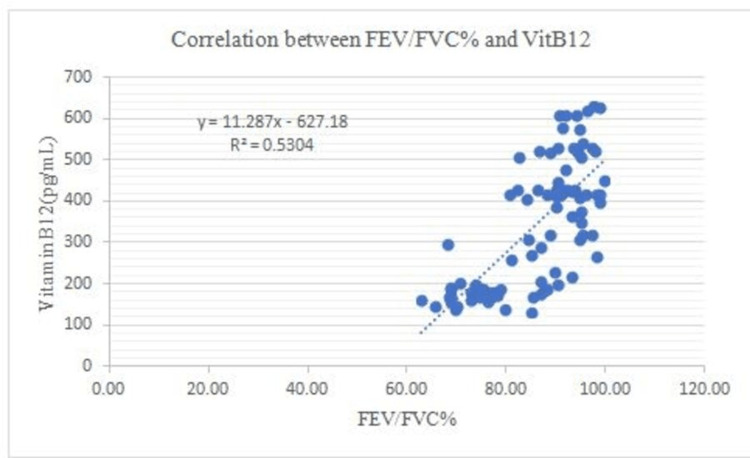
Correlation between FEV/FVC% and Vitamin B12 Shows a positive correlation between FEV/FVC% and Vit B12 FEV- Forced expiratory volume FVC- Forced vital capacity

There is a negative correlation between Vit B12 and HOMA-IR and in addition to this, FEV1: FVC% correlates negatively with BMI (0.53), WHR (0.50), glucose (0.68), insulin (0.68), Insulin resistance (0.80), and a positive correlation between FEV1: FVC% and Vit B12 (0.73) (Figures 1-7).

## Discussion

We aimed to explore the cardiovascular risk as assessed by insulin resistance and vitamin B12 in pediatric asthma which are poorly controlled because subclinical Vitamin B12 deficiency is quite common in our population. It is an often ignored entity in the management of several diseases which may require attention to prevent future devastating complications [13].

In our study, serum levels of vitamin B12 were low and inversely related to lung function in poorly controlled asthma. In concordance of our finding a recent study showed improvement in asthma symptoms after parenteral B12 [14]. A recent study in the Canadian population used serum vitamin B12 as a biomarker along with C-reactive protein (CRP) for lung function [15]. Likewise, in our study, serum B12 is comparatively low in poorly controlled asthma and negatively associated with lung function tests as predicted by FEV1% and its ratios (Table 1, Figure 3). A meta-analysis by Tea Skaaby documented that levels of vitamin B12 and folate were causally related to asthma [16,17]. Both B12 and folic acid, interdependently involved in ‘one Carbon metabolism’ donates methyl groups in various biochemical reactions, epigenetic modification by DNA methylation reactions can affect the genetic expression. Literature suggests that supplementation of B complex vitamins reduces cardiovascular diseases [18]. We didn’t measure the folic acid levels because of economic constraints, which depend on B12 for methyl tetra hydro folate reductase, the key enzyme involved in the production of the active form of folate remains a limitation of the study.

Insulin resistance is raising globally and is involved in several clinical inflammatory conditions affecting metabolism. Still, the impact of insulin resistance, and hyperinsulinemia on lung function is poorly understood ie hypoxic mediated insulin resistance. In our study, We found increased insulin resistance in poorly controlled asthmatics who had low vitamin B12 status. A study states that excess insulin can directly affect lung cellular physiology, which provides a clue of a similar molecular mechanism between asthma and cardiometabolic syndrome. Insulin resistance modified the association between asthma and obesity. The effect of obesity on asthma is more on individuals with insulin resistance [19]. Our study showed an increase in BMI and WHR in poorly controlled than well-controlled asthma in comparison to healthy subjects and an inverse relationship with lung function tests. This is in concordance with earlier reports i.e., asthma is more severe in obese individuals. Few studies have shown increased weight gain in asthma kids [20].

The current study shows hyperinsulinemia and Insulin resistance in poorly controlled asthma. This is due to hypoxia-induced stress, inflammation, and increased glucose level which can be due to recurrent oral steroid or salbutamol Beta 2 receptor agonist therapy and reduced physical activity. A study has shown in women asthmatics, stress and quality of life are affected more than male patients [21,22].

The study also documented insulin resistance in lung tissues, which can affect immunity, leading to repeated infection. This can trigger or exaggerate asthma episodes. A possible mechanism for immune dysfunction due to insulin resistance and B12 deficiency is a continuous release of histamine from the decarboxylation of histidine from mast cells through Th2 type mediated intracellular insulin signaling pathways like phosphoinositol 3 kinase (PI3K) which prevents smooth muscle relaxation of bronchus [19,23]. 

The limitation of the study includes a small sample size, and lack of dietary data of the study participants as it may affect the vitamin B12 values. 

## Conclusions

This study concludes that the level of B12 is decreased and insulin resistance is increased in poorly controlled asthmatic children in comparison to well-controlled asthma. These factors, along with the increased BMI in poorly controlled asthma, can predispose to cardiometabolic events. There is a positive correlation between the pulmonary function test and vitamin B12 and a negative correlation between the pulmonary function test and BMI and insulin resistance. Low levels of serum B12 were associated with insulin resistance and obesity in pediatric asthma. Thereby, this study suggests vitamin B12 supplementation and correction of insulin resistance in the management of asthma in the pediatric population.
